# Transmission of Donor-Derived* Trypanosoma cruzi* and Subsequent Development of Chagas Disease in a Lung Transplant Recipient

**DOI:** 10.1155/2017/5381072

**Published:** 2017-08-21

**Authors:** A. B. Corey, D. Sonetti, J. D. Maloney, S. P. Montgomery, B. L. Rademacher, L. J. Taylor, J. A. Smith, R. Striker

**Affiliations:** ^1^Division of Infectious Disease, Department of Medicine, Medical College of Wisconsin, 9200 West Wisconsin Avenue, Milwaukee, WI 53226, USA; ^2^Division of Pulmonary and Critical Care, Department of Medicine, University of Wisconsin, UW Med Centennial Building, 1685 Highland Ave., Madison, WI 53705, USA; ^3^Division of Cardiothoracic Surgery, Section of Thoracic Surgery, Department of Surgery, University of Wisconsin, 600 Highland Ave., MC 3236, Madison, WI 53792, USA; ^4^Parasitic Diseases Branch, Centers for Disease Control and Prevention, 1600 Clifton Road, MS A-06, Atlanta, GA 30329, USA; ^5^Division of General Surgery, Department of Surgery, University of Wisconsin, 600 Highland Ave., Madison, WI 53792, USA; ^6^Division of Infectious Disease, Department of Medicine, University of Wisconsin, Microbial Sciences Building, 1550 Linden Dr., Madison, WI 53706, USA

## Abstract

Donor infection status should be considered when accepting an organ for transplant. Here we present a case of Chagas disease developing after a lung transplant where the donor was known to be* Trypanosoma cruzi* antibody positive. The recipient developed acute* Trypanosoma cruzi* infection with reactivation after treatment. Chagas disease-positive donors are likely to be encountered in the United States; donor targeted screening is needed to guide decisions regarding organ transplant and posttransplant monitoring.

## 1. Introduction

Chagas disease, caused by infection with the parasite* Trypanosoma cruzi*, is endemic to many regions of Latin America and represents the largest burden of disease due to a parasitic infection in the Western Hemisphere [1]. Acquisition most commonly occurs by contact with an infected triatomine bug but can also occur via vertical transmission, blood transfusion, and organ transplantation. Infection can result in severe gastrointestinal or cardiac disease. While progress has been made in decreasing vector-borne transmission in some endemic regions, an estimated 6-7 million people remain infected throughout Mexico and Central and South America [2].

Chronic* T. cruzi* infection persists indefinitely in the absence of treatment. Approximately 70% to 80% of chronically infected individuals have the indeterminate form of infection and exhibit no signs or symptoms [3]. However, consequences of infection in the setting of immunosuppression may be deadly and appropriate management requires specialized interdisciplinary care [4]. Currently, two antitrypanosomal drugs have proven efficacy against Chagas disease; neither drug is approved by the US Food and Drug Administration (FDA) but both are available in the United States under investigational protocols through the Centers for Disease Control and Prevention (CDC) [3, 5].

Transplant-associated transmission has been reported in the United States since the early 2000s [4, 6]. The “Chagas in Transplant Working Group” has provided recommendations for screening of Chagas disease in organ donors and transplant recipients [7]. Previous reports suggest that the risk of transmission is dependent upon organ type and that transplantation of liver and kidneys from seropositive donors may be feasible [6]. However, evidence to support recommendations for other organs is lacking. We report a case and complete follow-up of* T. cruzi* transmission via bilateral lung transplantation from a seropositive donor.

## 2. Case Summary

The donor was a 48-year-old woman originally from El Salvador with history of heart failure and arrhythmias who died from a stroke. Testing for* T. cruzi *antibodies was pursued due to prior residence in an endemic area and cardiac history, and results were positive. Infection status was known at the time of organ donation and both lungs and liver were harvested. The liver recipient expired 11 weeks after transplant from septic shock following retransplantation after primary nonfunction of the graft from the* T. cruzi *infected donor. Posttransplant monitoring in that recipient revealed no evidence of* T. cruzi* transmission [6].

The lung recipient was a 36-year-old man who had been hospitalized due to severe pulmonary exacerbation of end-stage cystic fibrosis. During hospitalization, his treatment course was complicated by hypercapnic respiratory failure requiring mechanical ventilation in the intensive care unit. Despite aggressive ventilatory support, he continued to decline and his pretransplant 30-day in-hospital mortality was estimated to be nearly 100%. His parents consented to bilateral lung transplantation after being counseled on the risks of donor-derived infection from receipt of transplant from a donor with chronic Chagas disease.

Induction immunosuppression included basiliximab and high dose dexamethasone; tacrolimus, mycophenolate mofetil, and prednisone were used for maintenance immunosuppression. Due to high risk of cytomegalovirus (CMV) transmission from the donor (donor positive, recipient negative), prophylaxis with valganciclovir and immunoglobulin was initiated.

**Figure 1:**
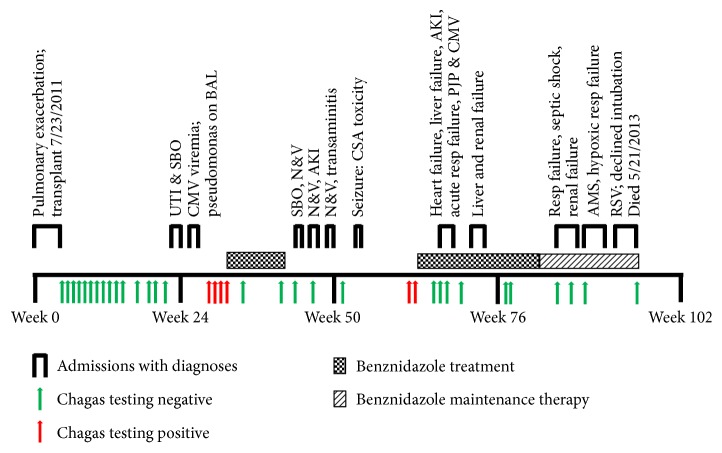
Time course of Chagas reoccurrences and complications after transplant. While it is unlikely that the patient would have survived his admission for respiratory failure without a transplant, a significant portion of this posttransplant life was marred by both* T. cruzi *detected in the blood and hospitalizations for various causes. Urinary tract infection (UTI), small bowel obstruction (SBO), bronchial alveolar lavage (BAL), acute kidney injury (AKI), cyclosporine (CSA), nausea & vomiting (N&V),* Pneumocystis jiroveci* pneumonia (PJP), respiratory failure (resp failure), altered mental status (AMS), and respiratory syncytial virus (RSV).

Immediately following transplant, Chagas disease surveillance was initiated based on the 2011 Chagas Working Group Guidelines [7] which recommended manual blood smear review and* T. cruzi *PCR weekly for the first 8 weeks after transplant, every other week for the next 8 weeks, monthly for the next 8 weeks (weeks 24–28 after transplant), and at any time immunosuppression is increased. In this patient, monitoring was done weekly for 9 weeks and then biweekly until 17 weeks after transplant; PCR and smear remained negative. During this time, the patient required multiple hospitalizations (see Figure 1) and modification of his immune suppression based on both therapeutic monitoring and the occurrence of infections.

*T. cruzi* monitoring was interrupted by hospitalizations and patient availability between week 17 and week 29. During week 29, routine monitoring was reinitiated and the patient tested positive by PCR, hemoculture, and serology (trypomastigote excreted-secreted antigen [TESA] immunoblot). He was clinically stable with no symptoms clearly referable to Chagas disease. Benznidazole was obtained from the CDC and treatment began at week 31 after transplant with 300 mg twice daily and continued for 60 days. Samples collected at week 34 were negative by PCR although serology remained positive. By week 43, two weeks after ending the 60-day course of benznidazole, both serologic and PCR testing were negative.

The patient developed several other transplant-related issues including transaminitis attributed to itraconazole, widely fluctuating tacrolimus levels, and worsening renal function. He was hospitalized for supratherapeutic tacrolimus complicated by acute psychosis and kidney injury. Tacrolimus was subsequently changed to cyclosporine and itraconazole was held. He continued to have elevated liver enzymes without clear etiology; a liver biopsy was nondiagnostic. He also developed candida esophagitis, pseudomonas pneumonia, and persistent low-level CMV viremia.

At 52 weeks after transplantation, the recipient returned to work part-time. However, he was hospitalized shortly thereafter following a seizure while driving. Workup revealed both cyclosporine toxicity and scarring from an old brain abscess. He was started on levetiracetam.

Routine* T. cruzi* monitoring was negative at weeks 47 and 51. The patient did not exhibit symptoms suggestive of Chagas disease, but* T. cruzi* PCR was positive at weeks 61 and 63 which prompted reinitiation of benznidazole. At week 66, the PCR was again negative. At week 67 however, the patient was hospitalized with fever, fatigue, dyspnea, orthopnea, and peripheral edema. Transthoracic echo demonstrated new onset dilated cardiomyopathy with ejection fraction .30–.35, grade 2 diastolic dysfunction, and global hypokinesia of the left ventricle—findings suggestive of Chagas myocarditis. There was no evidence of myocardial infiltration or scarring on cardiac MRI. Myocardial biopsy was performed but pathologic review and* T. cruzi* PCR were both negative. This hospitalization was also notable for ongoing transaminitis believed to be secondary to levetiracetam and congestive hepatopathy, as well as acute kidney injury resulting from cyclosporine and aggressive diuresis. The patient was also treated for hospital acquired pneumonia and* Pneumocystis jiroveci* pneumonia (PJP) diagnosed by PJP PCR from BAL. His immunosuppression was reduced because of the Chagas disease and opportunistic infections. Over the next few weeks the patient improved and was discharged. He was instructed to remain on the benznidazole for prophylaxis given the recurrence of likely parasitemia at 200 mg 3x/week.

The patient was readmitted twice in the ensuing months for pseudomonas otitis, sterile (*T. cruzi *PCR negative) pericardial effusion, respiratory failure, viral and pseudomonas pneumonia, altered mental status, and worsening kidney injury requiring hemodialysis. He was eventually diagnosed with chronic rejection/bronchiolitis obliterans and was treated with high dose steroids.

Following discharge, the patient continued prophylaxis and weekly monitoring for Chagas disease reactivation; test results continued to be negative. Approaching the two-year posttransplant mark, he was admitted to the hospital in respiratory failure with RSV pneumonia. He declined intubation and subsequently died 97 weeks after transplant.

## 3. Discussion

A growing shortage of donor organs has encouraged alternative efforts to expand organ supply. Using organs from donors with treatable infections offers a potential solution. For example, organs from hepatitis B-seropositive donors have been successfully transplanted into vaccinated recipients [8]. Use of organs from infected donors requires reliable and effective methods of monitoring for disease transmission as well as effective prophylaxis or treatment.* T. cruzi* infected donors may be an additional source of organs.

Although an unusual occurrence, transmission of* T. cruzi* via allograft has been previously reported in the United States for solid organs [4, 6]. Based on these limited reports, it appears that transmission is not universal and may depend on the type of organ [6]. Risk factors for transmission are not well identified. While heart transplantation from* T. cruzi* seropositive donors is currently not recommended due to higher transmission risk and tropism of the protozoan, previous reports suggest transplantation of liver and kidneys from infected donors may be considered [6, 9]. However, the safety of lung transplants from donors with Chagas disease is unclear.

This paper provides additional follow-up on the clinical outcome of a case of* T. cruzi *transmission by lung transplantation that has been previously reported [6]. Our patient was closely monitored for infection according to the Chagas Working Group Surveillance schedule and promptly treated with benznidazole for 60 days after infection was first detected. His reactivation was also promptly detected and treated and benznidazole was continued indefinitely. Of note, the patient was asymptomatic at the time of acute infection and when reactivation was first detected.* T. cruzi* PCR was negative at the time of death. The management of this patient is concordant with current recommendations against use of initial chemoprophylaxis and supports previous reports that suggest preemptive surveillance strategies can identify infection before the onset of symptoms.

Given the complex course for this patient, it is difficult to know exactly what contributed to this patient's death. Many infections have been associated with immunologic changes thought to favor the development of chronic allograft dysfunction [10]. Even though posttransplant treatment for* T. cruzi* appeared successful, this infection together with multiple other infectious complications led to modification of his immune suppression which likely contributed to the development of chronic allograft dysfunction. Thus, donor-derived* T. cruzi* infection may have been one factor that contributed to his death less than 2 years after transplant.

Previous case reports have suggested that transplantation of liver and kidneys from* T. cruzi *infected donors may be acceptable [6, 8]. However, lung transplant recipients typically require higher levels of immunosuppression than are needed for liver and kidney recipients and are therefore at heightened risk for donor-derived infections [11]. Further research is needed to estimate risk of transmission, determine optional management, and define posttransplant outcomes for lung transplant recipients who become infected with* T. cruzi*. In cases with high estimated pretransplant mortality, lung transplant from* T. cruzi* infected donors may be reasonable.

## References

[1] Bern C. (2015). Chagas' Disease. *The New England Journal of Medicine*.

[2] Chagas disease (American trypanosomiasis). World Health Organization, Geneva, Switzerland, 2016 http://www.who.int/mediacentre/factsheets/fs340/en/

[3] Bern C., Montgomery S. P., Herwaldt B. L. (2007). Evaluation and treatment of chagas disease in the United States: a systematic review. *Journal of the American Medical Association*.

[4] Kun H., Moore A., Mascola L. (2009). Transmission of Trypanosoma cruzi by heart transplantation. *Clinical Infectious Diseases*.

[5] Coura J. R., de Castro S. L. (2002). A critical review on chagas disease chemotherapy. *Memorias do Instituto Oswaldo Cruz*.

[6] Huprikar S., Bosserman E., Patel G. (2013). Donor-derived Trypanosoma cruzi infection in solid organ recipients in the United States, 2001-2011. *American Journal of Transplantation*.

[7] Chin-Hong P. V., Schwartz B. S., Bern C. (2011). Screening and treatment of chagas disease in organ transplant recipients in the United States: recommendations from the chagas in transplant working group. *American Journal of Transplantation*.

[8] Huprikar S., Danziger-Isakov L., Ahn J. (2015). Solid organ transplantation from hepatitis B virus-positive donors: consensus guidelines for recipient management. *American Journal of Transplantation*.

[9] Mccormack L., Quiñõnez E., Goldaracena N. (2012). Liver transplantation using chagas-infected donors in uninfected recipients: a single-center experience without prophylactic therapy. *American Journal of Transplantation*.

[10] Fisher C. E., Preiksaitis C. M., Lease E. D. (2016). Symptomatic respiratory virus infection and chronic lung allograft dysfunction. *Clinical Infectious Diseases*.

[11] Fishman J. A. (2007). Infection in solid-organ transplant recipients. *New England Journal of Medicine*.

